# Use of a structured functional evaluation process for independent medical evaluations of claimants presenting with disabling mental illness: rationale and design for a multi-center reliability study

**DOI:** 10.1186/s12888-016-0967-6

**Published:** 2016-07-29

**Authors:** Monica Bachmann, Wout de Boer, Stefan Schandelmaier, Andrea Leibold, Renato Marelli, Joerg Jeger, Ulrike Hoffmann-Richter, Ralph Mager, Heinz Schaad, Thomas Zumbrunn, Nicole Vogel, Oskar Bänziger, Jason W. Busse, Katrin Fischer, Regina Kunz

**Affiliations:** 1 Division Evidence-based Insurance Medicine, University Hospital Basel, Spitalstrasse 8 + 12, CH-4031 Basel, Switzerland; 2 Department Clinical Research, University of Basel, Spitalstrasse 8 + 12, CH-4031 Basel, Switzerland; 3 Basel Institute for Clinical Epidemiology & Biostatistics, University Hospital Basel, Basel, Switzerland; 4 School of Applied Psychology, Institute Humans in Complex Systems, Olten, Switzerland; 5 Swiss Society of Insurance Psychiatry, SGVP, Basel, Switzerland; 6 Institute of Medical Disability Evaluations of Central Switzerland, Lucerne, Switzerland; 7 Department of Insurance Psychiatry, Swiss Accident Insurance Fund, Lucerne, Switzerland; 8 Private practice for Psychiatry and Psychotherapy, Lucerne, Switzerland; 9 Psychiatric University Clinic of Basel, Basel, Switzerland; 10 Institute for Medical Disability Evaluation Interlaken, Lucerne, Switzerland; 11 Clinical Trial Unit, University Hospital Basel, Basel, Switzerland; 12 Zurich Office of the Swiss National Disability Insurance, Zurich, Switzerland; 13 Private practice, Wetzikon, Switzerland; 14 Department of Clinical Epidemiology and Biostatics, McMaster University, Hamilton, ON Canada; 15 The Michael G. DeGroote Institute for Pain Research and Care, McMaster University, Hamilton, ON Canada; 16 Department of Anesthesia, McMaster University, Hamilton, ON Canada

**Keywords:** Disability evaluation (MeSH), Work capacity evaluation (MeSH), Disability insurance (MeSH), Insurance Medicine (not MeSH), Reliability (not MeSH, related MeSH-term: reproducibility of results), Evidence-based medicine (MeSH), Mental disorders (MeSH), Psychiatry (MeSH), International Classification of Functioning, Disability and Health

## Abstract

**Background:**

Work capacity evaluations by independent medical experts are widely used to inform insurers whether injured or ill workers are capable of engaging in competitive employment. In many countries, evaluation processes lack a clearly structured approach, standardized instruments, and an explicit focus on claimants’ functional abilities. Evaluation of subjective complaints, such as mental illness, present additional challenges in the determination of work capacity. We have therefore developed a process for functional evaluation of claimants with mental disorders which complements usual psychiatric evaluation. Here we report the design of a study to measure the reliability of our approach in determining work capacity among patients with mental illness applying for disability benefits.

**Methods/Design:**

We will conduct a multi-center reliability study, in which 20 psychiatrists trained in our functional evaluation process will assess 30 claimants presenting with mental illness for eligibility to receive disability benefits [Reliability of Functional Evaluation in Psychiatry, RELY-study]. The functional evaluation process entails a five-step structured interview and a reporting instrument (Instrument of Functional Assessment in Psychiatry [IFAP]) to document the severity of work-related functional limitations. We will videotape all evaluations which will be viewed by three psychiatrists who will independently rate claimants’ functional limitations. Our primary outcome measure is the evaluation of claimant’s work capacity as a percentage (0 to 100 %), and our secondary outcomes are the 12 mental functions and 13 functional capacities assessed by the IFAP-instrument. Inter-rater reliability of four psychiatric experts will be explored using multilevel models to estimate the intraclass correlation coefficient (ICC). Additional analyses include subgroups according to mental disorder, the typicality of claimants, and claimant perceived fairness of the assessment process.

**Discussion:**

We hypothesize that a structured functional approach will show moderate reliability (ICC ≥ 0.6) of psychiatric evaluation of work capacity. Enrollment of actual claimants with mental disorders referred for evaluation by disability/accident insurers will increase the external validity of our findings. Finding moderate levels of reliability, we will continue with a randomized trial to test the reliability of a structured functional approach versus evaluation-as-usual.

## Background

Western countries have insurance systems in place that provide wage replacement benefits to individuals whose reduced health restricts or prevents them from working [1]. Over the last decade, most countries of the Organization for Economic Co-operation and Development (OECD) have experienced escalating rates of disabled workers, with current estimates ranging between 4 to 8 individuals per thousand of working age population per year [2]. In absolute terms, the number of new recipients of disability benefits per year ranges between 16,000 individuals for smaller countries like Switzerland and 1,700,000 for countries like the USA which constitutes a substantial economic challenge for society. Mental illness is currently the most common cause of disability from work [2–4].

**Fig. 1:**
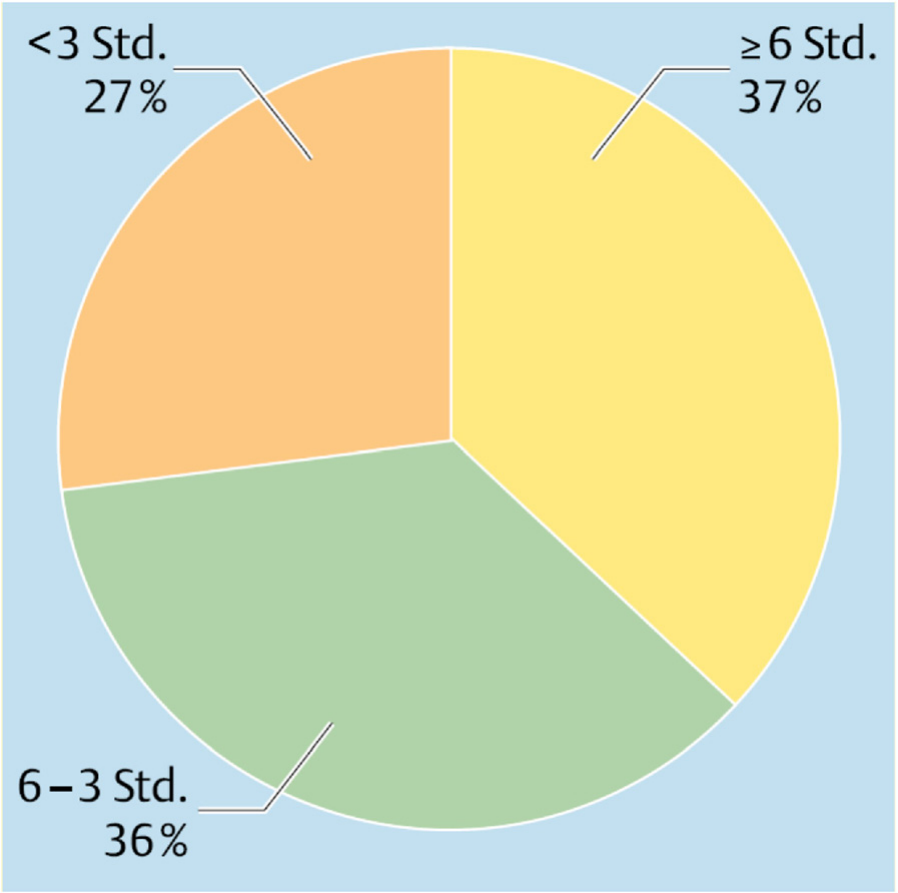
The case: A 49 year old female clerk with recurrent depressive disorders and a current episode of depression of moderate severity (ICD 10-diagnosis: F33.1) underwent a medical evaluation for disability benefits. The evaluation was videotaped and – together with the clerk’s medical notes - circulated to 22 psychiatric experts with the request to provide a medical diagnosis and a judgment of her work capacity in her previous job. ([9], with permission of the publisher, Licence number 3764760136993). The German disability benefit system allocates claimants for disability benefits in one of three categories: able to work more than six hours = full work capacity; able to work between three and six hours = partial work capacity; able to work less than three hours = unable to work

Both public and private insurance systems commonly use medical evaluations conducted by independent health professionals to determine the functional capacities of workers who claim inability to work due to illness or injury [5–7]. The findings of these independent disability evaluations are highly influential, often determining whether or not a claimant receives wage-replacement benefits, and the amount of compensation. Expectations prevail that claimants with similar health impairments and similar work demands will receive similar judgments regarding their (in-)ability to work from medical experts. However, anecdotal evidence suggests that many evaluation reports are of poor quality [8] and that different experts attending the same claimants often disagree regarding their ability to work (Fig. 1) [9–14].

A key criticism of disability evaluations is the failure of medical experts’ to clearly relate how claimants’ impaired health affects their ability to engage in competitive employment [14–18]. Rather, experts refer to their implicit professional expertise [11, 12, 19, 20]. This gap is prevalent in work capacity evaluations independent of the underlying health condition, and may be a fundamental source for variation between expert judgments and contribute to low reliability of work capacity evaluations.

**Fig. 2:**
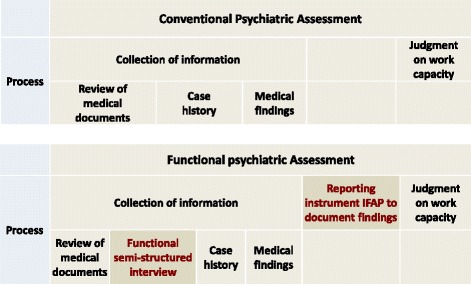
Functional evaluation integrated in the conventional psychiatric assessment which is performed according to the personal routine of the psychiatric expert

In an effort to improve transparency and reliability, we developed a multi-facetted functional approach to work capacity evaluation, with a focus on the claimants’ functional deficits and their remaining functional capacities. We call this approach “functional evaluation”. Functional evaluation complements conventional psychiatric evaluations that psychiatric experts perform according to their personal routine (Fig. 2). Since structure and standardization improve reliability [21–25], a structured approach for eliciting work-related functional information from the claimants and a standardized reporting instrument for documenting experts’ findings are central components of the functional evaluation.

The functional evaluation consists of a semi-structured interview with a focus on the claimants’ work, their self-perceived work (in-)capacity and their remaining ability to perform work-related tasks in relation to their health complaints; a detailed job description about the previous workplace obtained from the employer by the insurers and a selection of showcase jobs for suitable alternative work, and the Instrument for Functional Assessment in Psychiatry (IFAP) to document claimants’ functional capacities and limitations in common work-related activities [26].

**Fig. 3:**
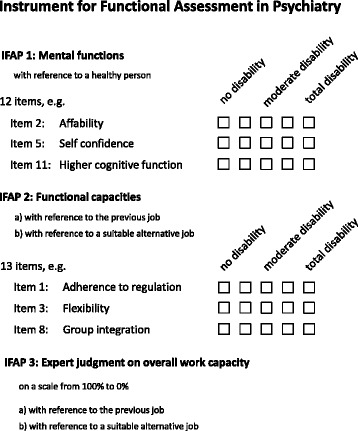
Structure of the Instrument of Functional Assessment in Psychiatry, IFAP

The IFAP uses a structured semi-quantitative format to report the functional capacity of claimants (Fig. 3). First, psychiatrists report twelve key mental functions which are commonly affected in claimants with mental disorders (IFAP 1): temperament and personality, affability, psychic stability, openness towards new experience, self-confidence, mental energy, attention, memory, emotional function, thinking, higher cognitive functions, experience of self and of time. Each function is defined by the International Classification of Functioning, Disability and Health (ICF) [27] and rated on a 0 (no impairment) to 4 (completely impaired) scale with anchor definitions for the impairment ratings.

**Fig. 4:**
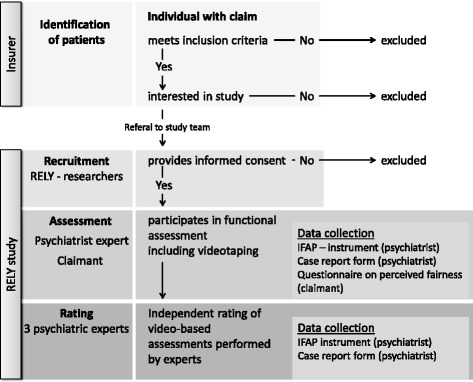
The RELY study. Recruitment and study flow

Second, the IFAP uses the Mini-ICF-APP [28] to report work-related functional capacities commonly impaired in claimants with mental disorders. The Mini-ICF-APP has been developed and validated in the German occupational rehabilitation setting [29–31]. It was recently translated and validated in a social psychiatry setting (single center secondary mental health care service) in the United Kingdom [32] and in a community mental health center in Italy [33]. The Mini-ICF-APP allows users to rate 13 work-related functional capacities: adherence to regulations, planning and structuring of tasks, flexibility, competency, endurance, assertiveness, contact with others, group integration, intimate relationships, non-work activities, self-care, mobility, and competence to judge and decide. Using a 0 (no limitations) to 4 (completely limited) scale, experts rate functional limitations associated with each item in reference to a detailed description of the previous job (IFAP 2a). To rate work ability in suitable alternative work, assessors select a reference job from a spectrum of showcase jobs in a large hotel which seems most suited to the claimants’ experience and remaining capacities, and rate in the same way any functional limitation for each of the 13-items in reference to the selected hotel job (IFAP 2b).

Finally, psychiatrists reflect on their ratings on the claimants’ mental functions and functional capacities. They estimate for how many hours the claimants should be able to work and their productivity before providing a judgment of the claimants overall work capacity on a scale from 100 to 0 % in the previous job and any suitable alternative job (IFAP 3). This judgment is used by disability insurers to calculate the amount of wage replacement benefits that will be paid to injured or ill workers through disability benefits.

The RELY-study [RELY stands for Reliability of Functional Evaluation in Psychiatry] will enroll patients with mental disorders claiming disability benefits and explore the inter-rater reliability of expert judgments regarding claimants’ work (in-) capacity based on a semi-structured functional interview and standardized reporting of work-related functional capacities. This study aims to demonstrate that the functional approach can achieve moderate reliability (ICC > 0.6) on work capacity evaluations in patients with mental disorders. The psychometric properties for the items of the three IFAP‐instruments (mental functions, activity limitations, work requirements) will show an interrater reliability above 0.5 for at least 85 % of the items, and above 0.65 for at least 20 % of the items. Justification of the thresholds: A recent study in social insurance addressing similar factors in claimants found that 6 raters evaluating ten items in twenty case vignettes reached an interrater reliability between 0.34 and 0.72 [34].

## Methods/Design

To optimize completeness and transparency in reporting the RELY study protocol, the structure and content of this manuscript was informed by the Guidelines for Reporting Reliability and Agreement Studies (GRRAS-guidelines) [35] Fig. 4.

### Study design

RELY will be a multi-center reliability study in which four psychiatric experts will independently assess workers undergoing a disability evaluation to assess restrictions and limitations associated with their mental illness.

### Setting

Consecutive claimants will be recruited from the Zürich office of the Swiss National Disability Insurance and from the Swiss National Accident Insurance Fund, Suva, while the evaluations and videotaping will be arranged through four independent assessments centers (two in Basel, one in Lucerne, and one in Interlaken).

### Participants

Medical and administrative staff at the insurance offices will identify and contact eligible claimants who have been scheduled to undergo a polydisciplinary disability evaluation, including a psychiatric evaluation. Eligible claimants will be workers whose first time disability evaluation includes a psychiatric interview, and capable of communicating in German without an interpreter. No other exclusion criteria apply.

After providing claimants with brief information about the study, the administrative staff will request permission to provide their name and telephone number to the research team so that they can be contacted about joining the RELY study. At that point, the insurer’s involvement in the study will end. Claimants who approve being contacted for the study will receive a detailed patient information that explains the study and its implications for the claimant in plain language. Next, a researcher will call to discuss the study with the claimants, to answer any questions that may have come up and to explain the informed consent form for participation. The claimants are then being asked to sign and return the informed consent form to the researcher. (For further details see Declarations, 2. Ethics approval and consent to participate).

### Interviewers and raters

Medical experts eligible for our study will be psychiatrists who currently provide disability evaluations for the Swiss National Disability Insurance or the Swiss National Accident Insurance acquired through a convenience sample of 20 psychiatric experts. No further eligibility criteria (such as level of expertise or years of experience) applied to reflect real life and prevent sampling error [2]. According to Swiss practice [36], the vast majority of experts are active in patient care. Participating psychiatrists will undergo a standardized, pre-piloted, training program for functional evaluation (see below [26]).

All psychiatric experts will conduct at least one videotaped disability evaluation and rate up to seven videotaped evaluations completed by their colleagues. They will be reimbursed for their time according to their usual rate. Psychiatrists and claimants will not be blinded to the study. Psychiatrists performing the evaluation will not be visible on the video, however, psychiatrists rating claimants shown in the videos may recognize voices of their colleagues.

### Training program in functional evaluation

Our functional evaluation approach focusses on the claimants’ health related functional limitations that relate to their ability to work, and complements psychiatrists’ conventional evaluation. The functional evaluation is consistent with the framework of the International Classification of Functioning, Disability and Health [27] and consists of the following components: 1) a semi-structured functional interview focusing on the claimants’ self-perceived limitations to work as a starting point for the evaluation; 2) a reporting instrument (IFAP) to document experts’ judgments with regards to the claimants’ mental functions (12 items) and their functional capacities (13 items); and 3) a detailed description of their last job and any suitable alternative work.

The training consists of written material describing the interviewing approach and information about the IFAP-instrument. Three face-to-face meetings lasting for three hours each will be used to discuss the functional evaluation with a methodological and a content expert based on cases from the experts’ own practice. Between training sessions, psychiatrists will be asked to use the functional evaluation with their own claimants and bring forward to the meetings any questions that arise. Additional details of the functional evaluation training program are described elsewhere [26].

### Procedures

#### The interview

The assessment centers will assign all enrolled study claimants to the next available study psychiatrist. Following a review of the claimants’ medical records provided by the insurer, experts will use the functional evaluation technique [26] to interview claimants. Interviews are anticipated to last 2 to 3 h, and will take place at an assessment center or the psychiatrist’s office.

The same research assistant will record all videos to ensure standardized recordings, using two cameras to prevent technical failures. Claimants will be filmed, but the assessing psychiatrist will remain off camera. The interviewing experts will write a psychiatric evaluation report for the insurer which will be integrated in the final polydisciplinary report. Psychiatrists who conduct the evaluation interview will provide a summary of the claimant’s socio-medical history for the rating psychiatrists who will review the video of the evaluation and complete study forms as detailed below.

Following the interview, and in the absence of the expert, the research assistant will provide the Questionnaire on Perceived Fairness to all claimants, with instructions that they can complete and return the form immediately, or later at home and return it to the research center in a sealed envelope.

The ratings: Three psychiatrist raters from a pool of 20 raters will be randomly allocated, via a web application, to each enrolled claimant. Randomization will prevent selection bias (e.g., the same three raters consistently forming a group [‘rater-group-membership’]) [37]. To avoid contamination, raters will be blinded to co-raters who view the same videotape [38, 39]. Four ratings will be generated for each enrolled claimant: one by their interviewing psychiatrist and three by psychiatrists who will independently review the video of the evaluation.

Psychiatrist raters will review videos of claimants’ evaluation interviews, their medical histories and job descriptions, and complete the IFAP-instruments through a secured website. All psychiatrists will receive information on how to access the secured website and standby support from the research team in case of unforeseen challenges with the data-security system.

### Data collection

Sociodemographic data on all eligible claimants (age, gender, civil status, nationalities and country of origin) and psychiatric experts (age, gender) will be recorded, complemented by psychiatrists’ professional experience (years since specialization; years working as psychiatric expert; number of work capacity evaluations over the last year; working concurrently as treating psychiatrist and psychiatric expert [Yes/No]). For eligible claimants who chose not to participate, insurance staff will record their mental disorder, gender and age.

Following each disability evaluation, the interviewing psychiatrist and three psychiatric raters will record all mental disorders that impact on work capacity using ICD 10 coding. They will fill in the IFAP-instrument, thereby providing estimates and judgments on overall work capacity related to the claimants’ previous job and to suitable alternative work (single item on a scale from 100 to 0 %, higher values indicate better work capacity), and on the following variables: commonly affected mental functions (12 items on a 5-point scale, higher score indicate greater impairment) and functional capacities commonly affected in mental disorders, also related to the claimants’ previous job and to suitable alternative work (13 items on a 5-point scale, higher scores indicate greater impairment).

Each psychiatrist who completes a disability evaluation will rate their certainty regarding the claimant’s overall work capacity (scale from 0 to 10, higher scores indicate greater certainty), the severity of the claimants’ mental disorder(s) compared to that of other claimants with similar mental disorders (scale from 0 to 10, higher scores indicate greater severity), and the extent to which claimants in the study represent ‘typical claimants’ referred for disability evaluation.

Six criteria capture ‘typicality’ in the context of our study: severity of mental disorders, complexity of diagnoses, impact of the claimants’ mental disorders on work capacity, work trajectory, spectrum of jobs and occupational activities, and education and professional training. For each criterion, respondents will relate the current claimant to other claimants assessed in their practice, report the frequency of evaluating such a claimant on a 3-item scale (seldom/neither seldom nor often/often) and generate a global judgment on the frequency of encountering such a claimant (same 3-item scale).

#### Perceived fairness of the evaluation process

The Questionnaire on Perceived Fairness of the evaluation process is based on a similar validated questionnaire used in the Dutch National Disability Insurance [40]. This instrument is comprised of 30 items on topics such as the experts’ perceived level of preparedness for the interview, their explanations about the encounter and the next steps following the interview, comprehensiveness of the interview, opportunities for the claimants to respond and ask questions, and attention and respectful demeanor towards the claimant, with response options on a 5-point scale (higher scores indicate higher agreement with the statement) and one item on the general satisfaction with the psychiatric evaluation (10-point scale, higher scores indicate higher satisfaction). Immediately following an evaluation, we pretested the questionnaire with 33 claimants for comprehension, length, ease to use, acceptance, and made modifications according to feedback, such as simplifying the wording of some questions [41].

#### Psychiatrists’ perceptions of the functional evaluation process

A semi-structured telephone interview will compile the psychiatrists’ perceptions on the functional evaluation. They will rate the usefulness of all three components of the functional evaluation for reaching a global judgment on claimants’ work capacity in the previous job and any suitable alternative work. They will report to what extent they integrated the functional interviewing (all parts of the interview/some parts, and listing those/none) and the various components of the IFAP-instrument (mental functions/functional capacities/descriptions of suitable alternative work/nothing) in their routine of work capacity evaluation outside of the study.

### Sample size

Our primary outcome is psychiatrists’ global rating of work capacity (expressed as percentage work capacity) in the previous job and in suitable alternative work. The intra-class correlation coefficient (ICC) should be at least 0.6 to be useful [42], which seems achievable in the insurance medicine context. [43, 44] With a sample size of 30 claimants, each evaluated by four raters, we expect to estimate an assumed ICC of at least 0.6 with a precision expressed as the width of a two sided 95 % confidence interval (CI) of ± 0.15 [45].

In an exploratory analysis, we will assess which variables – clinical (diagnoses), professional (psychiatrists’ characteristics) and demographic (claimants’ characteristics) – are associated with variation in the reliability of expert judgments, although we anticipate that these analyses will be underpowered to detect significant associations.

### Statistical analysis

We will describe psychiatrist and claimant characteristics with absolute and relative frequencies for categorical variables and with mean and standard deviation, or median and interquartile range, for continuous variables. To explore whether random allocation of psychiatrists to claimants was successful, i.e., producing low rater-group-membership, we will calculate the frequency of the same two, three or four psychiatrists being allocated to a claimant (‘rater-group-membership’) and calculate the Jaccard similarity coefficient of each pair of psychiatrists.

Each rating (rather than a consensus rating) will be used for our analysis. To estimate the means and associated 95 % CIs for the primary and secondary outcomes we will use mixed-effects models that take into account the crossed cluster design (i.e., each psychiatrist will assess several claimants and each claimant will be assessed by several psychiatrists) by including crossed random intercepts for claimants and psychiatrists, if appropriate, in order to obtain adjusted standard errors [46].

Furthermore, we will use mixed-effects models to estimate the variance components needed for the computation of the ICCs of work capacity, IFAP items and IFAP item sums (i.e., the residual variance and between-claimant variance for the ICC_absolute agreement_, and additionally between-psychiatrist variance for the ICC_consistency_) [47]. The associated measures of precision (95 % CIs) will be obtained by model-based parametric bootstrapping. Additionally, we will perform exploratory subgroup analyses on the ICCs for work capacity for specific diagnostic groups (ICD 10 categories of mental disorders, F00-F09; F10-F19; F20-F29; F30-F39; F40-F48; F50-F59; F60-F69). The clinical interpretation of ICC and agreement will follow the criteria by Fleiss [48] and Cicchetti [49]. For the ICC: poor (ICC < 0.4); fair (0.40 to 0.59); good (0.60 to 0.74); excellent (0.75 to 1.00), for the level of percentage agreement: poor (<70 %); fair (70 % to 79); good (80 to 89 %); excellent (>90 %).

To assess the associations of certain claimant traits (age, gender, IFAP 2 scores) and psychiatrist judgments (severity of disease, certainty of rating) with the reliability of work capacity, we will use a likelihood ratio test to compare two mixed-effects models, with one model allowing for heteroscedasticity between the claimant groups as specified by the claimant trait or psychiatrist judgment of interest. We will compute ANOVAs of mixed-effects models with main diagnosis or severity as a fixed effect to assess differences in certainty in work capacity ratings between main diagnoses or severity of disease. To check for a rater effect with regard to work capacity, where some psychiatrists may be systematically more lenient or strict in their judgments, we will use a likelihood ratio test to compare the two corresponding mixed-effects models used for the computation of ICC_absolute agreement_ and ICC_consistency_.

#### Missing data

We will undertake various measures to ensure complete data collection, such as web-based data entry of IFAP-ratings, diagnostic codes and responses to the outcome questionnaire by the raters with enforced complete data entry prior to moving to the next page. We will conduct complete case analysis. If the proportion of missing data is higher than 10 %, we will resort to multiple imputation before conducting the planned analysis.

### Data protection and transparency

To establish trust in the study among patients and their representatives, we will establish a stakeholder group with members from patient organizations (*n* = 3), patient lawyers (*n* = 1), the academic legal profession (*n* = 1), cantonal and federal social courts (*n* = 2), professional medical societies (*n* = 3), and insurers (*n* = 3) whose names will be published on the study website. This group will have observer status and act as a guarantor for the RELY study which implies regular meetings (minimum once a year) for an update about study progress, public acceptance and awareness, interim and final results. Members can ask specific questions about content and course of the study and act as contact within their community although confidentiality about details of the meetings will be agreed upon. This level of transparency should support the study’s credibility to the public.

Furthermore, we will establish a study website to inform claimants and the public about the study purpose, design and organization, involved researchers, the members of the stakeholder group and funders.

## Discussion

### What this study contributes

Reliability studies are often used to evaluate the measurement properties of human observers [50]. The results of this real-life study will provide an estimate of the measurement properties among expert psychiatrists who use our functional evaluation approach when determining health-related work capacity of workers claiming disabling mental illness.

Insurer’s typically consider medical evaluations as the best evidence for informing whether or not to award disability benefits [14]. However, for any evaluation process to be valid, it must first demonstrate reliability (although reliable evaluations can still be invalid). Inter-rater reliability can inform about the fairness of expert judgments (i.e., whether the claimants‘ evaluators come up with similar levels of functional impairment and similar judgments on remaining work capacity) when an independent reference standard for the diagnosis ‘work incapacity’ is lacking. Reliability reflects the extent to which medical evaluations can discriminate between claimants despite measurement errors inherent in the assessment. Reliable evaluations should consistently distinguish between claimants with high, moderate and low work capacity when performed by knowledgeable experts.

### Multiple sources of variation contribute to unreliable evaluations

In work capacity evaluation, multiple sources of variation can reduce inter-rater reliability regarding the evaluation of mental functioning (IFAP 1) and functional capacities (IFAP 2) of claimants [51, 52]: Information variance when experts obtain different information from the claimants as a result of asking different questions; observation variance when experts differ in what they pick up and remember when presented with the same information; interpretation variance when experts attach different importance to what they observe and hear; and criterion variance when experts apply different criteria to score the same information. Furthermore, psychiatrists, who typically lack expertise in vocational evaluation, may vary in their interpretation of the consequences of the claimants’ functional limitations on job demands and work ability.

Table 1 summarizes our multi-facetted approach to reduce these potential sources of variability: the semi-structured functional interview on occupational limitations with its five steps, the reporting instrument with 13 items to document the claimants’ functional limitations in reference to a detailed job description, the anchor definitions for grading severity of functional limitations and the calibration with peers in small group case based-learning. Videotaping the interview with the claimant will protect against subject variance.

**Table 1:** Sources of variation creating unreliable evaluations and procedures to reduce variation (modified from [51])

Source of variation	Definition	How source of variation was addressed in the study	Anticipated impact of the study approach on reliability:
1. Information	Raters obtain different information as a result of asking different questions	Structured functional interview with 5 steps and typical questions	Supports experts to elicit similar information Anticipated impact: ++
2. Observation	Raters differ in what they notice and remember when presented with the same information	Reporting instrument for documenting functional findings with a five item scale for rating limitations and anchor definitions Detailed job description as currently used by the disability office, all items completed.	Indirect impact on observer variance: raters will elicit information during interview that allows them to fill in the reporting instrument. Direct impact on observer variance: raters all have identical information on the work place Anticipated impact: ++
3. Interpretation	Raters differ in the significance they attach to what is observed	Calibration during small group case-based learning	Calibration: Some impact during the training when experts discuss the significance of various findings; intervision / calibration Anticipated impact: ++ Videotaping may increase interpretation variance when the interviewer omits to elicit relevant information that raters would need to get a clear picture. Anticipated impact: − / - -
4. Criterion	Raters use different criteria to score the same information	Anchor definitions in the IFAP-instrument Job descriptions for hypothetical alternative work Training and calibration	Anchor definitions, explicit qualifiers, joint training calibration should exert a substantial impact Anticipated impact: ++ In work (in-) ability, the experts’ implicit criteria are often unknown
*5.* Subject	True differences exist in the subject between testing, e.g., when telling different things to different raters	Videotaping of evaluation interview	Videotaped interviews reduce subject variance. Anticipated impact: +++
6. Expert/Rater	● Raters differ in their understanding of job demands and the consequences of functional limitations for job performance; ● Differences in value framework impact on judgment of claimants’ ability to work	● Detailed job description as currently in use by the insurers, all items completed. Job descriptions for hypothetical alternative work ● Not addressed	Optimized real-life job descriptions (=all items completed) and provision of job descriptions for hypothetical alternative work will provide the same reference / benchmark to the expert Anticipated impact: ++

Legend: +small/++ moderate/+++ large impact on enhancing reliability; − small/-- moderate/--- large impact on reducing reliability

### Strengths

Studies on inter-rater reliability of expert judgments in disability evaluation are scarce. Considerable indirectness (e.g., the use of hypothetical patients [9, 13, 44, 53, 54] and experimental settings [55–57]) limits their generalizability to actual claimants. The RELY study will use functional evaluations from the real world to establish the inter-rater reliability of medical experts that apply our functional evaluation approach when judging work capacity. A careful analysis of the most important sources of variance affecting disability evaluation (Table 1 and [51, 52]) directed the development of appropriate techniques and instruments to reduce their impact on expert judgment.

The RELY study includes additional methodological safeguards to guard against bias [35, 58]. Consecutive claimants and a convenience sample of psychiatrists with an expected broad spectrum of expertise in medical evaluations will reduce sampling error [59]. Random allocation of three raters to each claimant will prevent group membership and bias. Four ratings per claimant (interviewer and three raters) will increase precision in estimate of the ICC [60]. Videotaping interviews will protect against subject variance where otherwise the claimant may report different things at different occasions. Raters will be blinded to co-raters to ensure independence of ratings, although complete success in blinding cannot be guaranteed. One outcome will assess the typicality of the study claimants and, if confirmed, will strengthen the generalizability of the findings.

### Limitations

Little is known about the impact of the experts’ personal value framework on the kind of efforts they expect from claimants and the level of endurance of strains they expect from claimants to tolerate at work [14, 52]. This potentially important source of variance is not considered in the RELY study.

In our study, we mandate that certain questions be asked but do not otherwise interfere with specific psychiatrist’s approach to disability evaluation. Experts coming from different schools of thoughts (e.g., systemic, behavioral, analytic approach) have developed individualized routines in performing psychiatric evaluations, which may present challenges to experts reviewing videotapes. Informal consultation with the participating psychiatrists revealed that experts should come to similar functional findings, independent of school of thoughts, and that the IFAP-instrument with its thirteen items on functional capacities would guide interviewers to explicitly probe around these work related items during the interview [52]. While reassuring, it remains to be seen whether the psychiatrists’ expectations about the low impact of school of thoughts on functional findings will hold.

Although the IFAP-instrument has not been validated in the evaluation of work disability, the core component – reporting of the functional capacities – has been validated in related settings, such as occupational rehabilitation [29, 30], or social psychiatry [32]. Furthermore the swift spontaneous uptake of Swiss psychiatric experts indicates a high degree of acceptance and face validity.

A major concern remains that high reliability and expert agreement does not necessarily ensure valid evaluations. More conceptual work is needed on how to determine the validity of expert judgments in disability evaluations [34, 52].

Little prior knowledge exists about the processes of usual psychiatric evaluations: How do experts sort the collected information? What are their implicit criteria to select certain information and discard others? What weights do they attribute to the information selected, which makes some information more relevant than others? An optimal research program on disability evaluation would have started by understanding the processes and their relative impact on the critical outcomes before studying the critical societal and patient related outcomes. This approach, however, would have lasted two decades and longer with uncertain results. We therefore used the experience and opinions from our expert group to fill the many gaps we encountered in the design of the study, fully aware of the importance but also limitations of expert advice.

While we expect the sample size to have enough power for measuring the pre-specified reliability with pre-specified precision, it will lack power to detect significant effects when analyzing the evaluation process and predictors for work capacity. These analyses will therefore only inform about trends, they may, however, help in the planning of future studies. Multiple imputation assumes that data are missing at random, and the complete case analysis assumes missing completely at random. Missing data may not be missing at random, a condition that no statistical analysis can address.

Limiting the study to German speaking claimants will exclude many migrants with insufficient language skills who represent a substantial proportion of people applying for work disability.

### Placing the RELY study in context

The RELY study addresses a narrow but important aspect of work disability: Structured exploration of claimant-reported work limitations and standardised reporting of findings about mental functions (IFAP 1) and functional capacities (IFAP 2). Many other aspects impact on variance and reliability of disability evaluation and therefore qualify as high priority topics for research [14, 61]. For example, we need a better understanding on how experts come to their highly complex judgments of an individual’s work ability, and decision-making in complex situations has evolved as a distinct area of research [62–64]. What kind of claimant information do experts use in their judgments and in what way? How do experts weigh single cues, and how do they aggregate information into a final judgment? What is the experts’ understanding of the functional demands of jobs available in the free labor market? To what degree are they aware of any mismatch between functional job demands and claimants’ functional limitations and how do they integrate such mismatch in their judgment on work ability? How to make the experts’ personal value framework on what to reasonably expect from claimants more explicit? How to minimize the impact of the experts’ personal expectations on the judgment of work capacity and replace them by explicit expectations that result from a societal consensus [65].

## Conclusion

The functional approach to assess work disability is a plausible approach to improve reliability among psychiatrists. Finding moderate levels of inter-rater agreement, we will encourage us to continue with a randomized trial to test the reliability of a structured functional approach versus evaluation-as-usual.

## Abbreviations

CI, confidence interval; GRRAS-Guidelines, Guidelines for Reporting Reliability and Agreement Studies; ICC, intraclass correlation coefficient; ICD, International Classification of Disease, Disability and Health; ICF, International Classification of Functioning, Disability and Health; IFAP-instrument, Instrument of Functional Assessment in Psychiatry; Mini-ICF-APP, Mini-ICF-APP Social Functioning Scale; RELY-study, Reliable Disability Evaluation in Psychiatry; Suva, Swiss Accident Insurance Fund
